# Optic Disk Pit with Sudden Central Visual Field Scotoma

**DOI:** 10.1155/2016/1423481

**Published:** 2016-10-31

**Authors:** Nikol Panou, Demetrios G. Vavvas

**Affiliations:** ^1^Ophthalmic Clinic, General Oncologic Hospital “Agioi Anargyroi”, Kaliftaki, N. Kifissia, 14564 Attiki, Greece; ^2^Monte J. Wallace Ophthalmology Chair in Retina, Boston, MA, USA; ^3^Harvard Medical School, Boston, MA, USA; ^4^Ocular Regenerative Medical Institute, Massachusetts Eye and Ear Infirmary and Massachusetts General Hospital, 243 Charles St., Boston, MA 02114, USA; ^5^Angiogenesis Laboratory, Massachusetts Eye and Ear Infirmary and Massachusetts General Hospital, 243 Charles St., Boston, MA 02114, USA

## Abstract

*Purpose*. To describe a case of optic disk pit (ODP) with sudden central visual field scotoma.* Methods*. A 49-year-old woman presented, reporting sudden painless central visual field loss 3 months prior to presentation. Neuroophthalmologic, systematic, and laboratory evaluation and full imaging processes were performed.* Results*. Fundoscopy and color photography demonstrated an optic disk pit inferotemporally. Perimetry identified central visual field horizontal scotoma. OCT revealed absence of serous retinal detachment, but disclosed inner retina thinning corresponding to the area of the visual field loss. Fluorescein angiography demonstrated delay in the cilioretinal arteries and also disclosed a relative delay in the perfusion of an arterial branch off the inferior retinal arcade. Clinical and laboratory evaluations were negative for any related pathology.* Conclusion*. Sudden central visual field scotoma in patients with ODP may be associated with delayed vascular filling of CRA and retinal arterioles within the optic disc anomaly region.

## 1. Introduction

Optic nerve pits have originally been described by Wiethe in 1882 as congenital excavations of the optic nerve [1]. In 1958, Petersen [2] reported that congenital optic nerve pits may be complicated by serous maculopathy. In 1978, Radius et al. noted the appearance of acquired optic pits in the progress of open-angle glaucoma [3]. Both congenital and acquired pits can be associated with visual field defects.

## 2. Case Presentation

**Figure 1:**
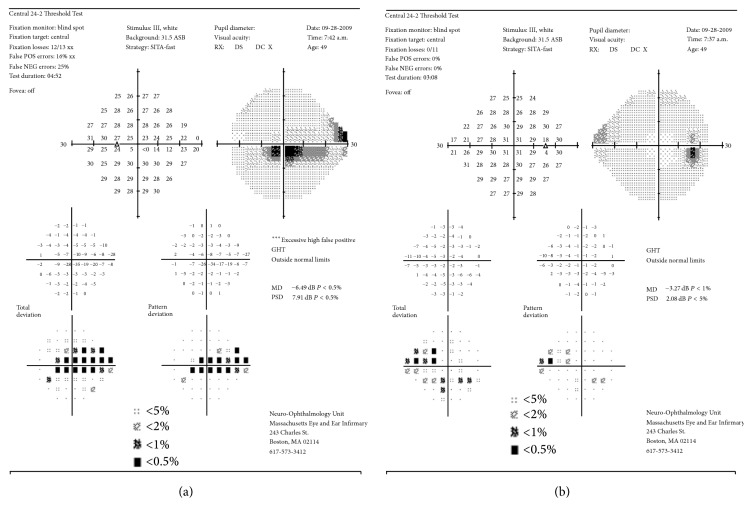
24-2 Threshold Test, Humphrey Visual Field Analysis. Central scotoma of the left eye (a) and the right eye (b).

**Figure 2:**
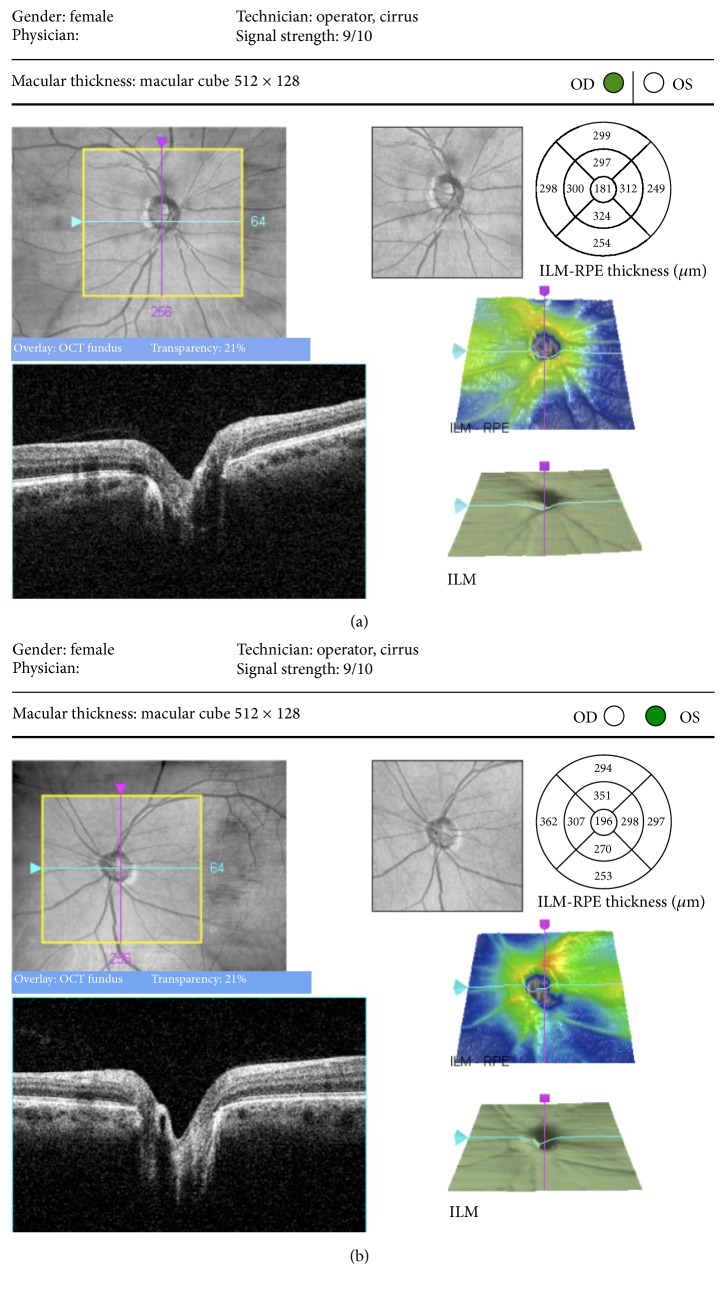
Spectral domain OCT centered on the optic nerve. (a) Right eye. (b) Left eye showing nerve fiber loss inferotemporally of the optic disk.

**Figure 3:**
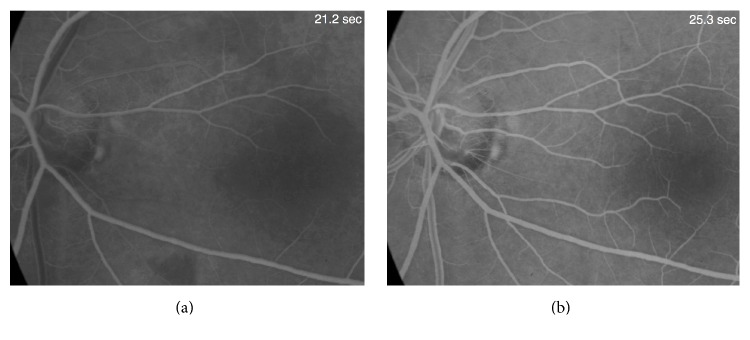
Fluorescein angiography showing delay of cilioretinal artery filling and a relative delay of the inferior branch retinal arteriole. (a) Filling of the retinal arterial system but not of the cilioretinal artery by 21.2 seconds. (b) Full filling of the CRA at 25.3 seconds.

A 49-year-old woman presented, reporting annoying, horizontal, bar-like positive scotoma, which suddenly appeared 3 months ago on the left eye. The visual acuity overall had remained stable. Neuroophthalmologic as well as systemic and laboratory evaluation failed to reveal any evidence of related pathology. A full ophthalmic imaging process was performed. Humphrey 24-2 visual field examination demonstrated central, horizontal visual field scotoma (Figure 1). The intraocular pressure was normal and the same in both eyes. Fundus examination and color photography revealed ODP inferotemporally, associated with a dark nerve fiber area. OCT confirmed absence of serous retinal detachment, but focal nerve fiber loss in correspondence with the dark nerve fiber area (Figure 2). The CRA was not filled until 25.3 seconds after injection (Figure 3). In addition, a delay in the perfusion of an arterial branch off the inferior retinal arcade was noted. There was a corresponding area of inner retinal thinning on OCT over the area of vascular filling delay.

## 3. Discussion

Usually patients with congenital optic pits may remain asymptomatic until complicated by serous macular schisis and detachment in their 30s or 40s [4]. In this case, no subretinal fluid schisis or detachment was identified. There is a single small series study by Adelung et al. [5] describing scotomas in patients with optic disk pit without subretinal fluid, also accompanied by defect in the nerve fiber layer, but no evidence of vascular blood flow as detected by FA was reported.

Glaucomatous visual field loss close to fixation in acquired pits of the optic nerve has been more frequently associated with lower pressures [6]. Our patient's visual field defect did not have glaucomatous characteristics and for this reason a vascular cause was investigated via fluorescein angiography. FA demonstrated delayed vascular filling in the area of OCT thickness loss and corresponding visual field loss. Vascular occlusion has not been previously reported in conjunction with optic disc pit, although optic disk pits have been associated with retinal venous anastomoses [7].
